# Molecular Dynamics Simulations of Binary Phosphate Glass Using the ReaxFF Potential

**DOI:** 10.1021/acs.jpcb.4c04925

**Published:** 2024-11-12

**Authors:** Zohreh Fallah, Jamieson K. Christie

**Affiliations:** Department of Materials, Loughborough University, Loughborough LE11 3TU, U.K.

## Abstract

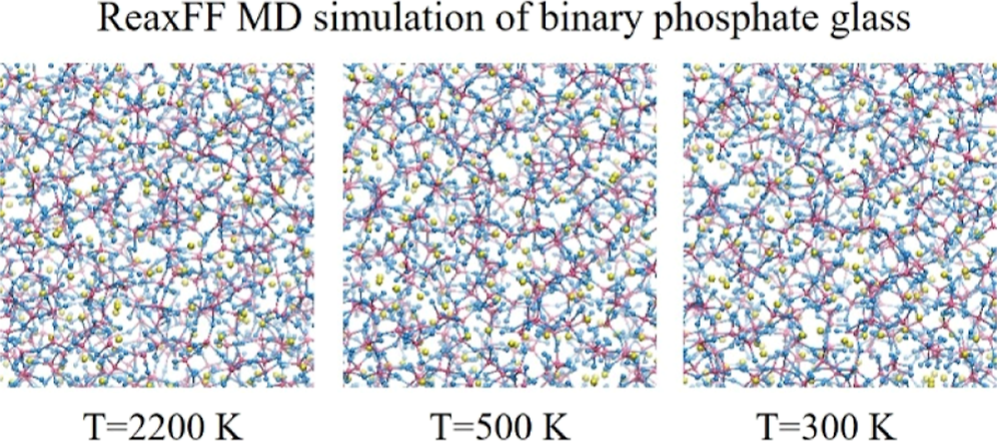

Due to the importance of the understanding of dissolution behavior of phosphate-based bioglasses (PBGs) in different biomedical applications, binary sodium and calcium phosphate glasses have been simulated for the first time using a newly developed ReaxFF force field and a standard melt-quench method with the LAMMPS classical molecular dynamics software. The partial radial distribution function of P–O within the first coordination shell indicated two distinct peaks corresponding to phosphorus bonding to NBO and BO, respectively, at distances consistent with those observed experimentally and a P–O coordination number of 4.0. Angular distribution functions were consistent with the experimental data. The calculated network connectivities are in good agreement with experimental data, and the detailed Q_*n*_ distributions are broader than would be expected.

## 1 Introduction

Phosphate-based glasses (PGs) have broad applications in optics, nuclear waste encapsulation, and biomedical applications depending on the composition and inclusion of therapeutic ions.^1−7^ The use of PGs in many of these applications is governed by their dissolution rate, which can vary by several orders of magnitude, depending on composition.^8,9^ It would therefore be very useful to understand the factors which control the dissolution of the glass to allow it to be optimized for specific applications. PGs have an amorphous atomic structure with the PO_4_ tetrahedron as a basic building block. Adding alkali ions depolymerizes the glass network and decreases the connectivity of the glass by forming nonbridging oxygen atoms (NBOs or terminal oxygens), which facilitates the dissolution of the glass in an aqueous environment. The number *n* of P–O–P linkages per PO_4_ tetrahedron is measured by the Q_*n*_ distribution, and the average value of *n* is known as the network connectivity (NC). This is a particularly important parameter as the NC is known to correlate with bioactivity^10,11^ and dissolution rate.

Computer simulation has been a powerful complement to experiment in understanding glass structure and properties,^12^ including PGs.^13^ Most previous work has focused on the structure of the glass alone,^14−18^ but of course dissolution is a product of the reaction between the glass and its environment, which occurs at the surface of the glass. Using computational techniques like ab initio and classical molecular dynamics (MD) simulations, we could investigate the interface of the glass and further understand the dissolution behavior of the glasses in aqueous environments. Due to the large computational expense of quantum-mechanical simulations, classical MD could be more useful, provided that an accurate interatomic force field (potential) can be found. There are different works focusing on the development of classical potentials to study PGs including a rigid-ion partial-charge force field employing a Lennard-Jones two-body potential,^19,20^ a rigid-ion Teter potential employing a Buckingham two-body potential,^21^ and a formal-charge polarizable force field including polarizability via the shell model (SM).^16,22^ But these classical potentials cannot show formation and dissociation of the chemical bonds; therefore, we need to use reactive potentials like Tersoff,^23^ REBO,^24^ BOP,^25^ AIREBO,^26^ and ReaxFF^27^ to investigate chemical reactions happening when the glass is immersed in an aqueous environment.

We have previously developed^28^ a ReaxFF potential for modeling the structure of PGs and showed that it well represents the structure of ternary glasses (Na_2_O–CaO–P_2_O_5_) such as those used for biomedical applications. In this work, we investigate the use of our potential to study binary phosphate glasses. Specifically, we investigate the structural properties of two types of binary phosphate glasses, sodium phosphate and calcium phosphate glass, each at two different compositions, (Na_2_O)_*x*_–(P_2_O_5_)_1–*x*_*x* = 0.5 and 0.379 and (CaO)_*x*_–(P_2_O_5_)_1–*x*_*x* = 0.5 and 0.6. This work is only concerned with the structure and properties of the bulk phosphate glass, and a future work will discuss the interaction of the glass with water.

There has already been substantial experimental work on the structure of these binary glasses to which we can compare to. The metaphosphate glass compositions of (Na_2_O)_0.5_–(P_2_O_5_)_0.5_ (hereafter P50Na50) and (CaO)_0.5_–(P_2_O_5_)_0.5_ (hereafter P50Ca50) consist of long chains of Q_2_ units linked via the bonds between sodium or calcium and NBOs.^29^

Phosphorus is bonded to both nonbridging and bridging oxygen (BO) atoms as seen by, e.g., Hoppe et al., who studied sodium phosphate glasses in a neutron diffraction study: the average distance and coordination number (CN) of P-NBO in (Na_2_O)_0.379_–(P_2_O_5_)_0.621_ (hereafter P62Na38) are 1.47 Å and 1.59, respectively, and in P50Na50 are 1.48 Å and 2.00, respectively, and the average distance and CN of P-BO (BO atom) in P62Na38 are 1.60 Å and 2.37, respectively, and in P50Na50 are 1.61 Å and 2.00, respectively, which gives a total CN of 3.96 and 4.0 for these compositions of binary sodium phosphate glasses, respectively.^30^

The mean interatomic distance and CN of P–O in P50Ca50 have been reported to be 1.51 Å and 4, respectively, which for Ca–O are 2.14 and 5.66 with the cutoff of 2.69 Å, respectively.^31^ In another experimental study on P50Ca50, the mean distance and CN of P–O are 1.54 Å and 4.1, respectively, and of Ca–O are 2.37 Å and 5.4, respectively.^32^

The XRD analysis of metaphosphate binary glasses showed that the CN of Na for an average Na–O distance of 2.43 Å is 5.7 ± 0.4; wherein the CN of Ca for the average Ca–O distance of 2.39 Å is 7.0 ± 0.4.^33,34^ P NMR spectra showed prominent peaks corresponding to Q_2_ and Q_3_, with Q_2_ larger, for the binary phosphate glass of 40 mol % Na_2_O, and prominent peaks corresponding to Q_1_ and Q_2_, with Q_2_ larger, for the binary phosphate glass of 53 mol % Na_2_O.^33^

It has been shown from diffraction by Hoppe et al. that the P–O distance depends on the molar ratio composition of different binary phosphate glasses.^34^ Modifier cations bond to NBOs at low cation concentration until all of the modifiers are surrounded by NBO; at higher concentrations, a modified random network is created,^35^ and the glasses with (Na_2_O)_0.379_–(P_2_O_5_)_0.621_ (P62Na38) and (CaO)_0.4_–(P_2_O_5_)_0.6_ (P60Ca40) are ultraphosphate glass structure dominated by randomly linked Q_2_ and Q_3_ species.^29,33^

In this paper, we are using our ReaxFF potential^28^ to investigate the structural properties of different binary phosphate glasses to see if the potential is transferable and able to characterize bulk binary phosphate glasses. In Section 2, we describe the force field and simulation methods before showing the structure of the glasses and comparing them with experimental data in Section 3. In Section 4, we conclude and propose directions for future work.

## 2 Methods

### 2.1 ReaxFF Force Field

The ReaxFF potential is an empirical bond-order potential that computes the energy by calculating the atomic distance between all atom pairs with the polarization effect being applied through a geometry-dependent charge calculation scheme.^27,36^ The potential allows for the investigation of the formation and dissociation of the bonds at every MD time step. The total energy is the summation of different partial energies, described by the following equation
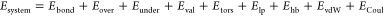
1where the partial energy terms present are bond energy (*E*_bond_), over- and under-coordination penalty energy (*E*_over_ and *E*_under_, respectively), valence angle energy (*E*_val_), torsion angle energy (*E*_tors_), lone-pair energy (*E*_lp_), hydrogen bonding energy (*E*_hb_), van der Waals energy (*E*_vdW_), and Coulomb energy (*E*_Coul_). If two atoms are not bonded, only the last two terms in eq 1 are nonzero.

Different sets of ReaxFF parameters are available from the literature to model binary phosphate glasses.^37−39^ Even by turning off the oxygen bond energy to avoid oxygen molecule formation at high temperature or doing ReaxFF MD only at room temperature while the melt-quench process is done using the polarizable core–SM, existing sets of parameters could not describe well the structural features of binary phosphate glasses like the CN of phosphorus and the NC of the glass. So, in this paper, we use our recently developed ReaxFF potential^28^ to investigate its transferability and ability for characterization of different binary phosphate glasses.

### 2.2 MD Simulation

Two different types of binary phosphate glasses at two different compositions have been selected which are (Na_2_O)_*x*_–(P_2_O_5_)_1–*x*_, with *x* = 0.5 and 0.379 and densities of 2.50 and 2.44 g/cm^3^, respectively,^30^ and (CaO)_*x*_–(P_2_O_5_)_1–*x*_ with *x* = 0.5 and 0.4 and densities of 2.611 and 2.557 g/cm^3^, respectively.^40^ These compositions have been chosen to allow a direct comparison to the experimental data. Five independent simulations with different initial configurations have been done for each composition, and the bond distance and other structural characteristics are the average from all five independent MD simulations. All MD simulations have been done with LAMMPS.^41^ The number of atoms in each composition is about 3000. The glass structures have been prepared using the melt-quench method, starting from heating up the random initial configuration at desired density at 2500 K for 100 ps using the core–SM.^22^ After this, the ReaxFF potential is employed at 2500 K, and the model is cooled to 300 K with a cooling rate of 5 K/ps and MD simulations at 300 K for 500 ps using the developed ReaxFF parameters (we also investigated performing the MD simulation of the whole process including the melting part that has also been done with the ReaxFF potential, and while some results were about the same, the Q_*n*_ distribution was inferior when compared to experiment.) The structural features have been calculated from the last 100 ps of ReaxFF simulation at *T* = 300 K. All simulations have been done in the *NVT* (constant volume and temperature) ensemble with the Nose–Hoover thermostat with a temperature damping constant of 20 fs and time step of 0.2 fs. Periodic boundary conditions in three dimensions have been applied in all simulations.

## 3 Results and Discussion

### 3.1 Short-Range Structure

Using the ReaxFF potential we previously developed, we simulated two types of binary phosphate glasses as mentioned above. We computed short-range structural properties including partial pair-distribution function, angular distribution function (ADF), and CN medium-range structural features such as NC and Q_*n*_ distribution of the glasses, where *n* represents the average number of BO in the first coordination shell of phosphorus.

**Figure 1:**
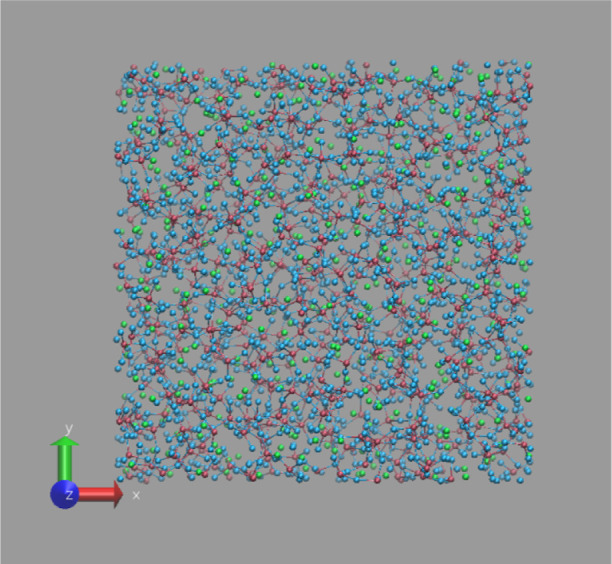
Snapshot of ReaxFF-simulated bulk PGs of P62Na38 at *T* = 300 K (color code: Na (green), O (blue), and P (dark pink)).

In Figure 1, the snapshot (one random frame in the last 100 ps out of 500 ps ReaxFF MD trajectory at *T* = 300 K) of the bulk binary phosphate glass of P62Na38 has been shown, where the box size is 34.64 Å.

**Figure 2:**
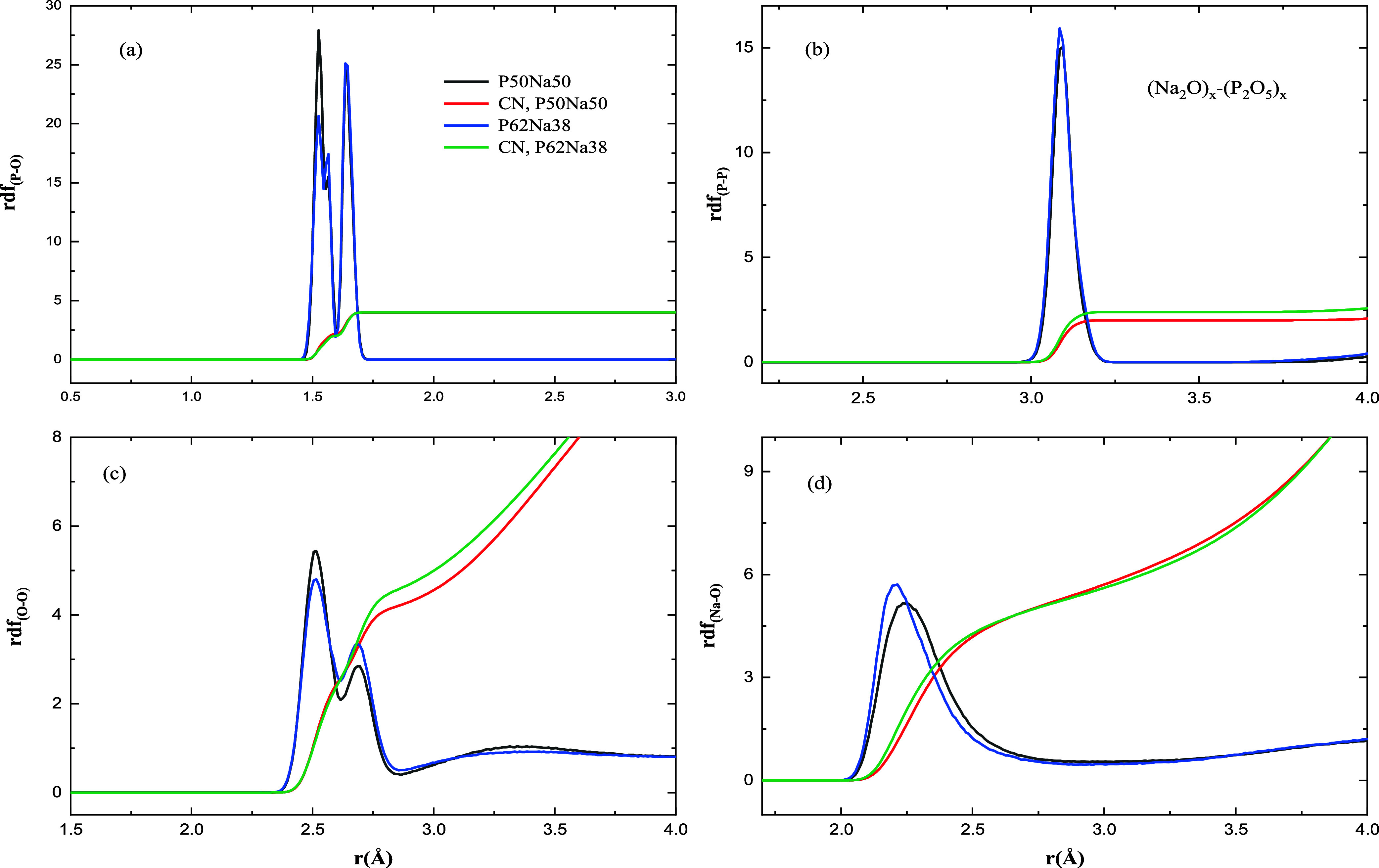
Partial RDF and CN in P50Na50 and P62Na38 at *T* = 300 K.

**Figure 3:**
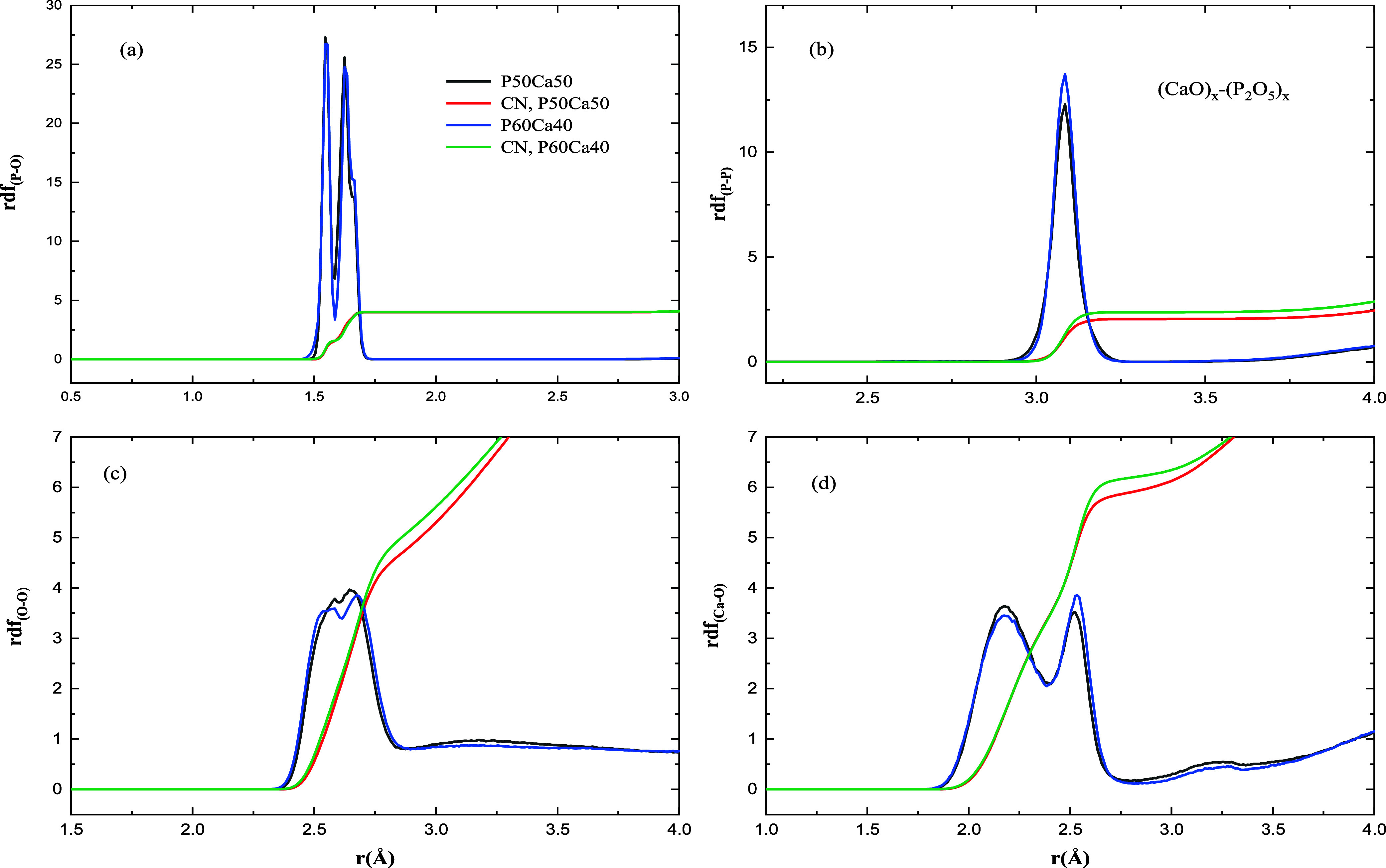
Partial RDF and CN in P50Ca50 and P60Ca40 at *T* = 300 K.

In Figures 2 and 3, different partial radial distribution functions (RDFs) of different binary phosphate glasses in P50Na50, P62Na38, P50Ca50, and P60Ca40 have been shown with the reported average distance of different atom pairs in Table 1. As shown in Figures 2a and 3a, there are two major peaks in the RDF of P–O representing bonding of phosphorus atoms to nonbridging (NBO) and BO atoms with the average bond distance of 1.53 and 1.64 Å in both compositions of P50Na50 and P62Na38, which are comparable with the experimental data, 1.48 and 1.61 Å in P50Na50 and 1.47 and 1.60 Å in P62Na39 for NBO and BO, respectively.^30^ There is a small shoulder (Figure 2a) around 1.57 Å in the RDF of P–O in both compositions of sodium PGs. It is not easy to assign a specific P–O bonding type for this shoulder, differentiating the bonding and making the P–O radial distribution function not symmetrical. The average bond distance of phosphorus to NBO and BO in both P50Ca50 and P60Ca40 is 1.55 and 1.63 Å, respectively, well comparable with the theoretical and experimental data.^31,32,35,42^ The averaged distance of phosphorus to phosphorus atoms in all compositions of binary glass is 3.08 Å, giving a single peak for the RDF of P–P (Figures 2b and 3b), which is in good agreement with experimental data.^31,42^

**Table 1:** Average Selected Distances (Å) and Angles (deg) of ReaxFF-Simulated Sodium PGs in Two Compositions of P50Na50 and P62Na38 and Calcium PGs in Two Compositions of P50Ca50 and P60Ca40 at *T* = 300 K

atomic pair	P50Na50	P62Na38	P50Ca50	P60Ca40
r(_P=_O)	1.53 Å	1.53 Å	1.55 Å	1.55 Å
r(P–O)	1.57 and 1.64 Å	1.57 and 1.64 Å	1.63 Å	1.63 Å
r(P–P)	3.08 Å	3.08 Å	3.08 Å	3.08 Å
r(O–O)	2.52 and 2.70 Å	2.52 and 2.69 Å	2.59 and 2.68 Å	2.59 and 2.65 Å
r(Na–O)	2.23 Å	2.23 Å		
r(Ca–O)			2.18 and 2.53 Å	2.18 and 2.53 Å
θ(O–P–O)	108.9°	108.9°	108.9°	108.9°
θ(P–O–P)	146.7°	146.7°	146.7°	146.7°
θ(O–Na–O)	60.3 and 89.1°	60.3 and 85.5°		
θ(O–Ca–O)			80.1 and 161.1°	77.5 and 157.5°

Similar with ternary phosphate glass,^28^ the RDF of oxygen–oxygen in both types of binary phosphate glass shows two peaks (Figures 2c and 3c). The first peak in sodium phosphate glass is around 2.52 Å, and the second one is around 2.70 Å in P50Na50 and around 2.69 Å in P62Na38, while the first peak in calcium phosphate glass is around 2.59 Å and the second peak is around 2.69 Å in P50Ca50 and around 2.65 Å in P60Ca40. It seems decreasing the modifier ions (Na^+^ or Ca^2+^) in binary phosphate glasses would make the second peak closer to the first one and gives a smoother distribution of O to O bond lengths.

**Figure 4:**
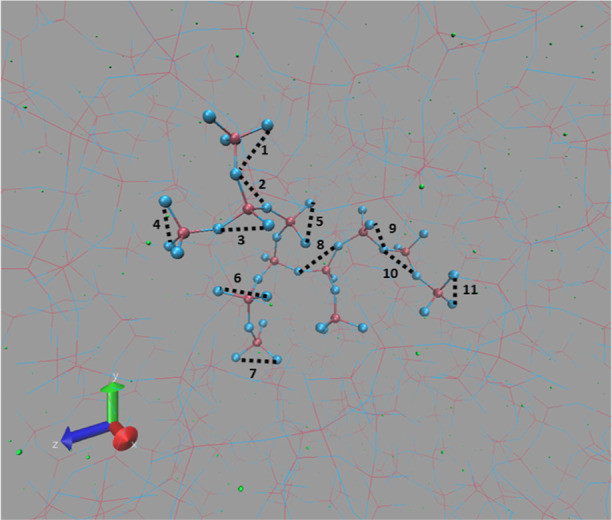
Different O–O distances numbered in one randomly selected segment of P50Na50 in simulated PG at *T* = 300 K (averaged distance with more details represented in Table 1).

To go into more details of two peaks in the RDF of O–O in sodium phosphate glasses, the distances between different types of oxygen (BO/NBO–BO/NBO) in one randomly selected segment of P50Na50 (Figure 4) have been averaged over the last 100 ns of 500 ns MD simulation at *T* = 300 K, represented in Table 2. Similar to ternary phosphate glass,^28^ the NBO–NBO distance is shorter than the BO–BO distance in the same phosphate group. As shown in Table 2, NBO–NBO in the Q_1_ group has the shortest distance and BO–BO in the Q_3_ group has the longest distance and NBO and BO in different Q_*n*_ groups have distances between them. On the other hand, NBO–NBO and NBO–BO in the Q_1_ group have shorter distance compared with NBO–NBO in the Q_2_ group and NBO–BO in Q_2_ and Q_3_ groups. It means that O–O distances in Q_1_ and some in Q_2_ groups would give the first peak, while O–O distances in Q_2_ and Q_3_ groups would give the second peak in the RDF of O–O. This is consistent with our understanding that P-NBO bonds are on average shorter than P-BO bonds, so units such as Q_1_ with a preponderance of NBOs will have shorter O–O bond distances.

**Table 2:** Averaged Oxygen–Oxygen Distances of P50Na50-Simulated PG Shown in Figure 4 and Averaged Calcium–Oxygen Distances of P50Ca50-Simulated PG Shown in Figure 5; Averaged Over the Last 100 ps of 500 ps *NVT* Simulation at *T* = 300 K

numbers in Figure 4	O–O specification	average distance (Å)	numbers in Figure 5	Ca–O specification	average distance (Å)
1	NBO–BO in Q_1_	2.53	1	Ca–NBO in Q_1_	2.60
2	BO–BO in Q_3_	2.74	2	Ca–NBO in Q_2_	2.04
3	NBO–BO in Q_3_	2.65	3	Ca–NBO in Q_0_	2.54
4	NBO–NBO in Q_1_	2.50	4	Ca–NBO in Q_1_	2.56
5	NBO–NBO in Q_2_	2.69	5	Ca–NBO in Q_2_	2.17
6	NBO–NBO in Q_2_	2.68	6	Ca–NBO in Q_2_	2.56
7	NBO–NBO in Q_1_	2.50			
8	BO–BO in Q_3_	2.82			
9	NBO–BO in Q_2_	2.67			
10	BO–BO in Q_2_	2.53			
11	NBO–NBO in Q_1_	2.51			

**Figure 5:**
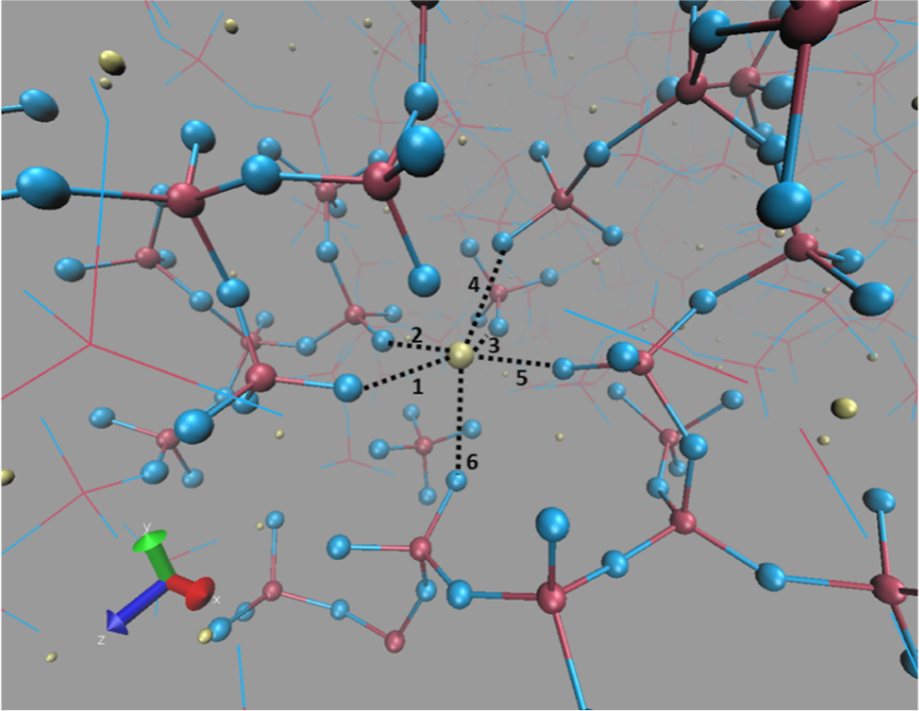
Ca–NBO numbered distances in different Q_*n*_ species in a randomly selected region of simulated P50Ca50 at T = 300 K (averaged distance with more details represented in Table 1).

The RDF of Na to O atoms shows a single peak around 2.23 Å (Figure 2d), well comparable with an averaged Na–O distance of 2.24 Å,^43^ 43 Å,^33^ and 2.38 Å^34^ in experimental works. The RDF of Ca to O atoms shows two peaks around 2.18 and 2.53 Å in binary calcium phosphate glasses, as shown in Figure 3d and Table 1. The distance between one representative calcium ion and its surrounding oxygen atoms in P50Ca50 has been averaged over the last 100 ns of 500 ns simulation at *T* = 300 K, shown in Figure 5 and represented in Table 2. Similar to ternary phosphate glasses,^28^ it seems calcium ions have shorter distance with NBO in Q_2_ species and longer distance with oxygen atoms in Q_0_ and Q_1_ species leading to two peaks in the RDF of Ca–O. The results are consistent with experimental data of the Ca–O averaged distance of 2.37 Å^32^ as well as reported two peaks for the RDF of Ca–O in binary calcium phosphate glasses by Wetherall et al.^42^

The CN is the integration of the corresponding RDFs up to its first minimum, as reported in Table 3. The total CN of phosphorus to oxygen atoms in the first coordination shell (using a cutoff of 2 Å) in four compositions of binary sodium glasses is 4, which is in very good agreement with the experimental data.^30,31,34^ The CN of P–NBOs (P=O) of sodium phosphate glass decreased from 1.70 to 1.28 by decreasing the Na^+^ concentration, consistent with the experimental data.^30,34^

**Table 3:** CN of P and Na/Ca to O Atoms in Binary Sodium/Calcium Phosphate Glasses with the Cutoff up to First Minima in the Corresponding RDF (∼2.0 Å for *P*, ∼3.0 Å for Na and Ca)

atomic pair	P50Na50	P62Na38	P50Ca50	P60Ca40
P=O	1.70 ± 0.01	1.28 ± 0.01	1.53 ± 0.02	1.59 ± 0.01
P–O(total)	4.0 ± 0	4.0 ± 0	4.0 ± 0	4.0 ± 0
Na–O	5.61 ± 0.02	5.50 ± 0.04		
Ca–O			5.90 ± 0.01	6.18 ± 0.01

The CN of Na–O with the cutoff around 3 Å is 5.61 and 5.50 in P50Na50 and P62Na38, respectively, which is in good agreement with the reported CN of Na in binary phosphate glasses.^33^ As shown in Table 3, the CN of calcium in binary calcium phosphate glasses, which is 5.90 in P50Ca50 and 6.18 in P60Ca40, is higher than of the CN of sodium in binary sodium phosphate glass, which is in good agreement with reported experimental data.^31,32^ As a whole, the ReaxFF potential is able to characterize the structural properties of different binary phosphate glasses well comparable with experimental data.

**Figure 6:**
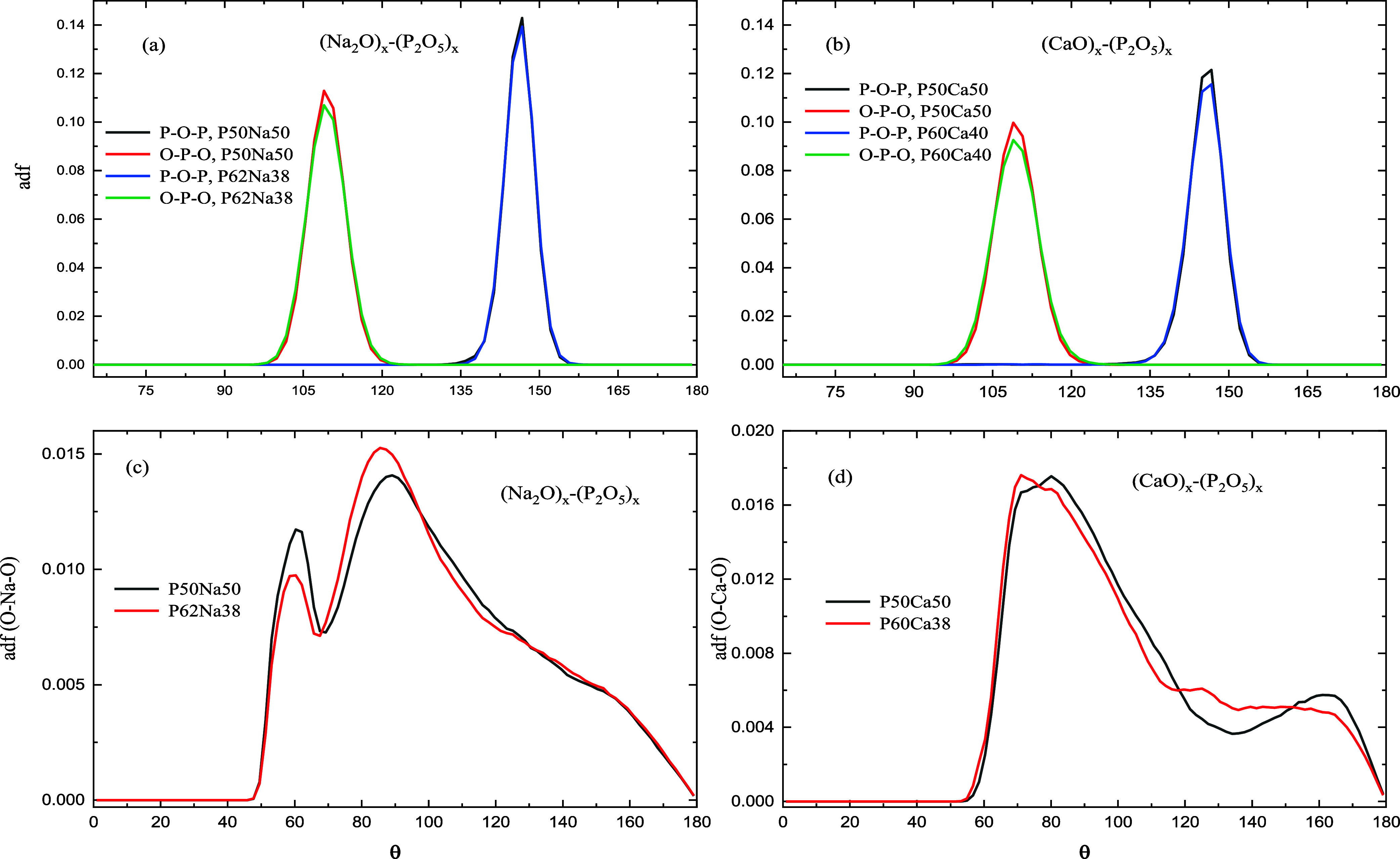
Different angular distribution function (adf) of bulk simulated, (a,c) sodium PGs in two different compositions P50Na50 and P62Na38, and (b,d) calcium PGs in two different compositions P50Ca50 and P60Na40 at *T* = 300 K.

The ADF in different simulated binary glasses is shown in Figure 6a–d. Three-body angles of P–O–P with the peak around 146.7°, of O–P–O with the peak around 108.9°, as well as angular distribution of O–Na–O with two peaks around 60.3 and 87.3° (averaged in both compositions) in binary sodium and O–Ca–O with one broad peak around 78.8° and one small peak around 159.3° (averaged in both compositions) are in good agreement with other theoretical and experimental data.^30,42,43^ The peak at about 60° in O–Na–O is due to the increased intratetrahedral bonding, where two oxygen atoms are part of the same PO_4_ tetrahedron, compared to O–Ca–O where the peak is not visible.^14,17^

### 3.2 Medium-Range Structure

The medium-range structural properties have been analyzed as these are particularly important to get right; as above, the NC is often used as a single parameter to describe the bioactivity of a specific glass composition. Q_*n*_ distribution and NC calculations have been presented in Table 4 showing the broad distribution of different species in both binary sodium and calcium phosphate glasses. As shown in Table 4, there are different Q_*n*_ species from *n* = 0 to *n* = 4 in simulated binary phosphate glasses. The NC of four compositions is in very good agreement with the theoretical and experimental data.^33,44,45^ The simulated NC of P50Na50, P62Na38, P50Ca50, and P60Ca40 is 2.01, 2.39, 2.06, and 2.35, respectively, with the error of approximately less than 0.003. It denotes that the ReaxFF parameters are able to model binary sodium phosphate glasses well. Although the Q_*n*_ distribution of bulk simulated binary glasses is in less good agreement with the experimental data,^33,45−47^ there are some points of agreement, e.g., Q_2_ is dominant in all four compositions as appropriate. The percentage of Q_2_ is higher in P50Na50 than in P62Na38, and the percentage of Q_3_ in P50Na50 is less than in P62Na38, and the percentage of Q_2_ species is higher than that of Q_3_ in P62Na38; all these trends are in agreement with the experiments and the statistical mechanical methodology being represented by Bødker et al. to predict the Q_*n*_ distribution of binary PGs.^44^ Similar trends have been observed for binary calcium phosphate glasses, as Q_2_ has the highest percentage in both compositions and is more in P50Ca50 compared with P60Ca40. Comparing sodium and calcium phosphate glasses, it seems Q_2_ is more abundant in calcium glasses, while Q_3_ is almost the same and Q_0_, Q_1_, and Q_4_ are a little more in sodium glasses, i.e., sodium phosphate glasses have more wider distribution of Q_*n*_ distribution compared with calcium phosphate glasses, as was found for ternary phosphate glasses.^28^

**Table 4:** Q_*n*_ Distribution (%) and NC of Sodium/Calcium PGs, Each in Two Compositions, Compared with the Theoretical Corresponded NCs

	*Q*_0_	*Q*_1_	*Q*_2_	*Q*_3_	*Q*_4_	NC	NC (theory)
P50Na50	3.43 ± 0.09	27.8 ± 0.46	38.5 ± 0.97	24.6 ± 0.51	5.67 ± 0.30	2.01 ± 0	2.0
P62Na38	0.76 ± 0.08	17.0 ± 0.25	36.23 ± 0.75	34.06 ± 0.64	11.94 ± 0.23	2.39 ± 0	2.39
P50Ca50	3.65 ± 0.29	21.53 ± 0.63	44.91 ± 0.99	25.48 ± 0.56	4.43 ± 0.16	2.06 ± 0	2.0
P60Ca40	1.61 ± 0.27	13.17 ± 0.51	41.31 ± 0.33	35.11 ± 0.22	8.80 ± 0.21	2.35 ± 0	2.33

## 4 Conclusions

Different compositions of binary phosphate glasses have been simulated for the first time using our recently developed ReaxFF potential to see if the force field is able to characterize the structural properties of different binary phosphate glasses to give us the opportunity for possible future investigation of dissolution behavior of glasses. The ReaxFF potential gives bond lengths and angles in good agreement with experimental data in both sodium and calcium binary phosphate glasses, as well as correct CNs and NC, although the Q_*n*_ distribution is broader than that found experimentally. We conclude that our potential is promising for the simulation of binary as well as ternary phosphate glasses.
